# Profile of non-tuberculous mycobacteria amongst tuberculosis presumptive people in Cameroon

**DOI:** 10.1186/s12866-024-03256-x

**Published:** 2024-03-26

**Authors:** Valerie Flore Donkeng Donfack, Thierry Anicet Zemo Fokou, Lazare Eric Noche Wadje, Yves Le Grand Napa Tchuedji, Yvonne Josiane Djieugoue, Sorelle Nguimfack Teagho, Brenda Shile Takong, Yannick Patrick Assolo, Suzanne Magloire Ongboulal, Yannick Willy Kamdem Simo, Stanley Nkemnji Awungafac, Sara Eyangoh

**Affiliations:** 1 Mycobacteriology Unit, National Tuberculosis Reference Laboratory, Centre Pasteur du Cameroun, Yaounde, Cameroon; 2 Department of Microbiology, University of Yaounde I, Yaounde, Cameroon; 3 Department of Microbiology, Haematology and Infectious Diseases, Faculty of Medicine and Biomedical Sciences, University of Yaounde I, Yaounde, Cameroon; 4 Centre Pasteur du Cameroun, Yaounde, Cameroon

**Keywords:** Profile, Non-tuberculous mycobacteria, Tuberculosis, Comorbidities, Tuberculosis presumptive people

## Abstract

**Background:**

Cameroon is a tuberculosis (TB) burden country with a 12% positivity among TB presumptive cases. Of the presumptive cases with a negative TB test, some are infected with Non-tuberculous Mycobacteria (NTM). However, the diagnosis of NTM infections remains difficult due to the lack of tools in many laboratories, particularly in resource limited laboratories and remote setting. The present study was undertaken to determine NTM profile and associated comorbidities among TB presumptive people.

**Methods:**

A retrospective study was conducted from December 2018 to December 2019 in the Tuberculosis-National Reference Laboratory (TB-NRL) for Bacteriological analysis of samples and Jamot Hospital of Yaounde (JHY) for clinical evaluation of confirmed NTM patients.

We included in this study data of 5267 TB presumptive people previously diagnosed using three consecutive samples and having culture and SD Bioline results with or without Microscopy and reverse hybridization-based Line Probe Assay(LPA) results. The data on co-morbidities or history of people infected with NTM were then collected from the three participants with available clinical data.

**Results:**

We collected data of 5267 presumptive TB people. Among them, 3436 (65.24%), have a positive culture with 3200 (60.75%) isolates belong to *Mycobacterium tuberculosis* Complex (MBTC) and 236 (4.48%) to NTM. Our results showed that, 123 (52.11%) NTM were isolated from people with negative microscopy and 113 (47.88%) from people with positive microscopy. Among the 236 NTM, 108 (45.8%) isolates were identified using LPA. *M. fortuitum* was the most represented species (32.41%) followed by *M. intracellulare* (19.44%). Sputum had the highest proportion of NTM (56%), followed by bronchial aspirations (31%). The extra-pulmonary samples presented lower proportions of isolates compared to pulmonary samples. Some patients affected with NTM presented comorbidities as HIV infection, Pulmonary tuberculosis, Type 2 diabetes, Chronic bronchitis and Alveolar pneumonia.

**Conclusions:**

Our study showed the presence of NTM strains among presumptive TB people with a predominance of *M. fortuitum* and *M. intracellulare*. It is important to implement a surveillance system of NTM in TB burden country and also to develop a point-of-care test for NTM identification in limited-resource settings.

## Background

NTM include all mycobacteria other than the species belonging to the MBTC and *Mycobacterium leprae*. They are ubiquitous environmental but some species cause opportunistic infections in animals and humans [1]. The incidence of NTM-related death has increased worldwide [2]. The clinically most important species include *Mycobacterium abscessus*, *Mycobacterium avium* complex, *Mycobacterium kansasii*, *Mycobacterium malmoense* and *Mycobacterium xenopi*. Like *Mycobacterium tuberculosis* Complex (MBTC), NTM cause pulmonary and extra-pulmonary infections. The number of diseases caused by NTM has increased dramatically in HIV people [3]. The most common clinical forms of NTM infections are pulmonary infections followed by lymphatic and tissue damage and disseminated infections [4]. These infections have clinical and paraclinical similarities with infections caused by *Mycobacterium tuberculosis* species. This causes confusion in the diagnosis, representing a real problem to the care of patients, especially since the treatment differs from NTM to MBTC. Previous studies reported the emergence of NTM in others countries. The study of Shahraki et al., shows that among suspected multidrug-resistant TB patients who did not respond to 2–3 months of treatment with first-line anti-TB drugs, about 30% were actually infected with NTM [5].

Several authors have published data on the diagnosis, signs, symptoms and management of *Mycobacterium tuberculosis* infections. WHO regularly publishes up-to-date data on TB prevalence and incidence in most countries around the world and these countries, including Cameroon have a TB surveillance network [6]. Despite the serious infections caused by NTM and the emergence of resistant strains, WHO has not created a platform for NTM surveillance like the one for TB and the data on NTM infections are not sufficiently documented as those of *Mycobacterium tuberculosis*. In Cameroon, surveillance of mycobacterial infections does not include NTM infections and there are almost no studies highlighting the NTM species circulating in Cameroon and associated comorbidities. The aim of this work was to determine the frequency of NTM and associated comorbidities in presumptive TB people.

## Methods

### Study design and data collection

**Fig. 1:**
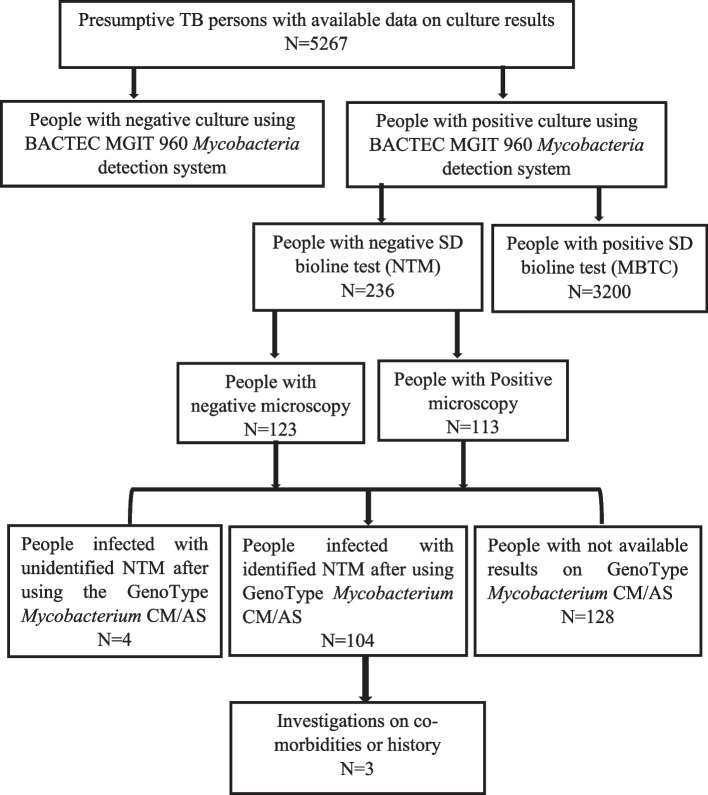
Flow diagram showing study population

A retrospective study was conducted from December 2018 to December 2019. Data from September 2016 to December 2019 on TB status were extracted from the registers in TB-NRL. We included in this study data of 5267 TB presumptive people previously diagnosed using three consecutive samples and having culture and SD Bioline results with or without Microscopy and reverse hybridization-based LPA (GenoType *Mycobacterium* CM and GenoType *Mycobacterium* AS) results. The data on comorbidities and history of people infected with NTM were then checked (Fig. 1).

### Study location

The study was conducted at the TB-NRL for Bacteriological analysis of samples and JHY for clinical evaluation of confirmed NTM patients.

The TB-NRL is located at the Mycobacteriology Unit of the Centre Pasteur du Cameroun (CPC). The TB-NRL have a technical platform for TB bacteriological analysis using WHO recommended rapid diagnosis (WRD), TB-LAMP and Xpert MTB/ RIF; BACTEC MGIT 960 for Culture and drug susceptibility testing (DST) and NTM bacteriological analysis using reverse hybridization-based LPA, GenoType *Mycobacterium* CM and GenoType *Mycobacterium* AS (Hain Lifescience, Nehren Germany). This laboratory received samples from presumptive TB and NTM cases coming from about 93 Diagnosis and Treatment Centers (DTC) from 03 regions of Cameroon (Centre, Est and South) for Culture and DST analysis.

The JHY is a reference hospital for pneumology and a DTC of Yaounde and its surrounding areas. JHY is also specialized in the management of patients infected with multi-drug-resistant *M. tuberculosis*. The hospital provides diagnostic tests for TB using sputum smear microscopy and TB-LAMP tests.

The CPC and Jamot hospital are in close vicinity and have a long history of collaborative research.

### Routine laboratory process

#### Microscopy

Microscopy was performed as described previously by Donfack et al. [7]. Sample was first smeared on a glass slide and fixation was conducted by heating on the fire. This was then flooded with auramine followed by adequate rinsing with water and discoloration with acid-alcohol solution (3% V/V). It was then rinsed well with water, stained with methylene blue (1% V/V), dryed and visualized under fluorescence microscopy.

#### TB-LAMP assay

The test was carried out with the HumaLoop T(Eiken Chemical Company Ltd, Japan) according to the WHO guideline [8]. The 60 µl sample was transferred to a heating tube and incubated at 90 °C for 5 min. After inactivation, the sample was mixed well with absorption powder and 30-μL DNA solution was extracted and placed in a reaction tube, and loop-mediated isothermal amplification was performed at 67 °C for 40 min. A positive control tube containing sputum with AFB and a negative control tube containing bacilli-free artificial sputum were used for each manipulation. Positive result was characterized by the presence of strong fluorescence in the tube, which was not released from the negative reaction.

#### Xpert MTB/RIF assay

The Xpert MTB/RIF assay was performed according to manufacturer’s instruction [9]. Sample reagent was added using a double volume of the sample, homogenized and incubated for 15 min at room temperature with mixing intervals. After inactivation, 2 ml of the treated sample was transferred to the cartridge containing all the reaction mixture and loaded to the GeneXpert instrument. An automatic process completed the remaining assay steps and the results were interpreted.

#### Culture

The culture was carried out using the automated system BACTEC MGIT 960 *Mycobacteria* detection system as recommended by the manufacturer. Samples were decontaminated in N-acetyl-l-cysteine-sodium hydroxide and 500 µL of the decontaminated sample was inoculated into a MGIT medium, incubated for 42 days in the MGIT™ 960™ instrument and the appearance of the colonies was then observed [10].

#### Identification of *Mycobacterium tuberculosis* complex

Immunological identification of isolates was done by rapid detection of MPT64 antigen with the immunochromatographic assay, (SD Bioline assay, Kyonggi, Korea). The SD Bioline assay was performed with acid fast bacilli in MGIT medium as described by the manufacturer [11]. A positive test confirmed the species of MBTC. All culture isolates which were negative for immunochromatographic assay were submitted for NTM identification.

#### Hybridization-based line probe assay

Culture isolates with a negative results for SD Bioline assay were processed first for primary identification using reverse hybridization-based line probe assay, GenoType *Mycobacterium* CM (Hain Lifescience, Nehren Germany), then the GenoType *Mycobacterium* AS for culture isolates which were negative to GenoType *Mycobacterium* CM. The process was performed as recommended by the manufacturer and was consisted of an extraction, amplification and hybridization [12].

#### Statistical analysis

Data was entered into the software Excel 2010 and validated by double entry and comparison. The frequencies were determined and the graphs were then plotted.

#### Ethics approval

This study was approved by the Cameroon National Ethics Committee for Human Health Research under the number N°2017/02/872/CE/CNERSH/SP. Patient identifying information was removed prior to analysis. As this was a study of routinely collected data, patient consent was not required.

## Results

We collected the data of 5267 presumptive TB people. Among these, 3436 have a positive culture (65.23%) of which 3200 (60.75%) isolates belonged to MBTC, 236 (4.48%) had a negative SD Bioline assay and were considered as NTM strains. Among these 236 NTM, 123 (52.11%) were isolated from people with negative microscopy and 113 (47.88%) from people with positive microscopy. The data on GenoType *Mycobacterium* CM and GenoType *Mycobacterium* AS of 128 (54.2%) isolates were not available and the data of 108 (45.8%) isolates were available of which 104 (96.29%) were identified. Of the 236 participants infected with NTM, sociodemographic data were available for 113 participants including 38.05% women and 61.94% men. The average age was 43.92 ± 19.30 years.

**Fig. 2:**
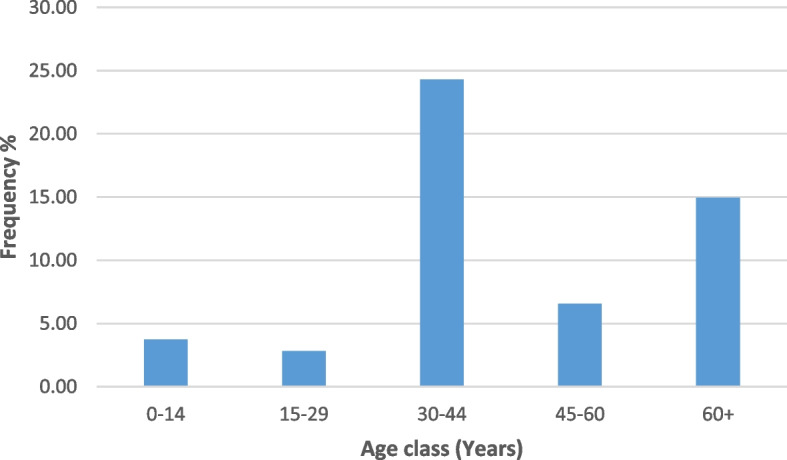
Distribution of NTM according to age class

Figure 2 showed the frequency of infected people according to the age class. Adults were the most infected (24.30%), followed by older adults (14.95%).

**Fig. 3:**
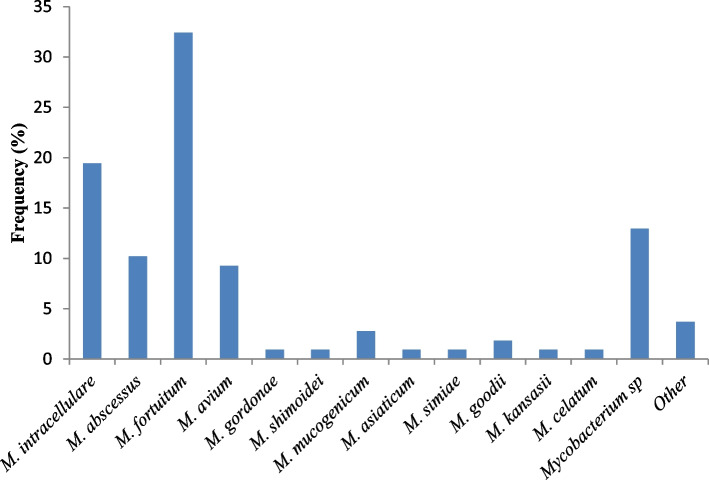
Frequency of species among NTM

Figure 3 shows the frequency of species among NTM. *M. fortuitum* was the most represented species (32.41%) followed by *M. intracellulare* (19.44%), *M. abscessus* (10.2%), *M. avium* (9.26%) and *Mycobacterium sp* (12.96%). Around 4 (3.7%) NTM were not identified with the LPA.

In this study 90.67% of NTM were isolated from pulmonary samples and 9.33% from extrapulmonary samples. Table 1 shows the proportion of NTM according to the samples. Sputum had the highest proportion of species with 60 NTM (56%), followed by bronchial aspirations with 34 NTM (31%), gastric tubing with 6 NTM (6%) and nasopharyngeal aspirations with 4 NTM (3%). On the other hand, the extra-pulmonary samples presented low proportions of isolates with frequencies ​​of 1(1%) for the stools, 1(1%) for the pus and 1(1%) for the biopsy.

**Table 1:** Frequency of NTM according to samples types

	**Stools** ***N* = 1**	**Bronchial aspiration** ***N* = 34**	**Nasopaharyngeal aspiration** ***N* = 4**	**Sputum** ***N* = 60**	**Broncho alveolar** ***N* = 1**	**Biopsy** ***N* = 1**	**Gastric tubing** ***N* = 6**	**Pus** ***N* = 1**
*M. Fortuitum*	0(0)	10(29.41)	0(0)	24(40)	0(0)	0(0)	1(16.66)	0(0)
*M. celatum*	0(0)	1(2.94)	0(0)	0(0)	0(0)	0(0)	0(0)	0(0)
*M. Kansasii*	0(0)	1(2.94)	0(0)	0(0)	0(0)	0(0)	0(0)	0(0)
*M. avium*	0(0)	4(11.76)	0(0)	3(5)	0(0)	0(0)	3(50)	0(0)
*M. intracellulare*	0(0)	6(11.64)	2(50)	12(20)	0(0)	0(0)	1(16.66)	0(0)
*M. mucogenicum*	0(0)	0(0)	0(0)	3(5)	0(0)	0(0)	0(0)	0(0)
*M. shimoidei*	0(0)	0(0)	0(0)	1(1.66)	0(0)	0(0)	0(0)	0(0)
*M. asiaticum*	0(0)	0(0)	0(0)	1(1.66)	0(0)	0(0)	0(0)	0(0)
*M.gordonae*	0(0)	0(0)	0(0)	2(3.33)	0(0)	0(0)	0(0)	0(0)
*M.simiae*	0(0)	0(0)	0(0)	0(0)	1(100)	0(0)	0(0)	0(0)
*M. abscessus*	1(100)	1(2.94)	2(50)	6(10)	0(0)	1(100)	0(0)	0(0)
*M.goodii*	0(0)	1(2.94)	0(0)	1(1.66)	0(0)	0(0)	0(0)	0(0)
*Mycobacterium. Spp*	0(0)	6(11.64)	0(0)	7(11.66)	0(0)	0(0)	1(16.66)	1(100)
Others	0(0)	4(11.76)	0(0)	0(0)	0(0)	0(0)	0(0)	0(0)

Among the people presenting NTM infections, data on medical history or comorbidities of only 3 participants were available. The three participants presented in the past respiratory diseases. The respiratory diseases were pulmonary tuberculosis in one patient, chronic bronchitis in the second, chronic bronchitis and alveolar pneumonia in the third. One of the infected participants was a person living with HIV and another had type 2 diabetes. The average age of these 3 patients was 47.3 years.

## Discussion

In Cameroon, the National Tuberculosis Control Program continuously monitors infections caused by *M. tuberculosis* and the resistance of these strains to anti-tuberculosis drugs. Distinguishing NTM from TB presumptive people is a major challenge, which requires sensitive test. In the present study, 236 (4.5%) people with positive cultures in the BACTEC MGIT 960 instrument who were initially presumed to have TB, were infected with NTM. These findings demonstrated that we need to find NTM strains in TB presumptive people who have a negative TB test. These results are lower than those obtained in northern India, which presented a NTM frequency of 29% between 2013 and 2015 [13]. The low frequency of NTM in our study may be due to the fact that the data were collected only from TB-NRL which is one of two laboratories which perform LPA for NTM identification in Cameroon. In addition, this low frequency could be also due to the lack of reagents, because 128 NTM strains out of 236 were not identified. The similarity of symptoms between TB and NTM infections can lead to overlooking of NTM infections. Microscopy which is the most widely used test in limited resource laboratories, does not distinguish NTM from MBTC. This is why it is necessary to have a good system for transporting samples from remote settings to the TB-NRL where LPA is available. In our study, 47, 88% NTM were isolated among sample with positive microscopy and 52.11% NTM were isolated among samples with negative microscopy although microscopy was repeated using three samples from each patient. These findings demonstrated that NTM-infected patients may be wrongly diagnosed in remote laboratory where only microscopy is used as initial diagnostic test for TB. Consequently, some patients infected with NTM and with negative microscopy would not be treated while some patients infected with NTM and with positive microscopy would be treated as TB patient. This can lead to unfavorable outcomes, increased morbidity and risk of mortality. Previous study conducted in Pakistan also demonstrated the isolation of NTM (6%) among people with negative microscopy [14]. Our findings demonstrated the diagnosis value of culture considered as gold standard, and highlight the usefulness of reverse hybridization-based line probe assay, GenoType *Mycobacterium* CM and GenoType *Mycobacterium* AS to diagnose NTM infections among presumptive TB people. However, these tests are expensive, require infrastructure and qualified staff. The development of a point of care test for NTM diagnosis will be helpful in laboratories with limited resources. Some countries like China revealed an increase in the prevalence of NTM strains from 4.3% in 1979 to 22.9% in 2010 through the surveillance system [15]. These findings demonstrates the need to monitor NTM infections in presumptive TB people with a negative TB test and the need to implement a surveillance system with a good transport system for samples from limited resource laboratories to the TB-NRL.

Our study showed that *M. fortuitum* was the most represented specie with a frequency of 32.41% followed by *M. intracellulare* with a frequency of 19.44%. The results obtained by Karamat et al. in Pakistan revealed that *M. avium* complex (55%) and *M. abscessus* (25%) were the most represented in pulmonary and extrapulmonary samples [14]. These findings showed that epidemiology of NTM infections in Cameroon differs from data obtained in other countries.

The largest proportion of strains was isolated from pulmonary samples with 56% isolated from sputum, followed by 31% isolated from bronchial aspirates (31%). These results are similar to those obtained by Gonzalo et al. in Uruguay [16], which found that pulmonary swabs had the highest proportions of NTM although the proportion of sputum was higher (66.6%) compared to the findings obtained in our study. Studies conducted by Ali et al. [17], in Iran also revealed that the highest frequency of NTM was found in pulmonary sample (27.1%).

The search for comorbidities was carried out only on three participants infected with NTM. This low number of participants is explained by the unavailability of clinical data in laboratories due to the lack of surveillance system of NTM in Cameroon. Among the people infected with NTM one was infected with HIV. The studies conducted by Surendra et al. in India also found that out of 42 patients infected with NTM, one patient was infected with HIV [18]. In our study, other comorbidities such as alveolar pneumonia, type 2 diabetes and pulmonary tuberculosis have also been identified in these people. Previous studies have revealed that in most cases, several factors such as the existence of a pulmonary diseases or immunosuppression promote infections by NTM [19]. In this study, chronic bronchitis was the most frequent comorbidity in these patients, in contrast to the studies conducted by Matesanz et al. which revealed that chronic obstructive pulmonary disease was the most frequent comorbidity in patients infected with NTM [20].

This study has several limitations. The low frequency of NTM is also attributed to the collection of data in the TB-NRL representing only data from 03 regions of the 10 regions of Cameroun.. All the NTM were not identified due to lack of reagents, and the absence of surveillance system of NTM in Cameroon do not allowed us to show the evolution of NTM infections. The unavailability of about 52.11% (123/236) of demographic data are also considered as a limitation of the study. Although the comorbidities have been identified, there is insufficient data to support the objective of NTM comorbidities in the study population. Further studies are necessary to extend the collection of clinical data on a large sample size to support the conclusion on NTM comorbidities.

## Conclusion

Our findings demonstrated that infections caused by NTM may be underdiagnosed in the absence of molecular tools as Hybridization-based LPA, GenoType *Mycobacterium* CM and GenoType *Mycobacterium* AS among presumptive TB people. It is important to implement a surveillance system of NTM including efficient sample transport system, to build staff capacity and upgrade existing diagnostic facilities across the country to ensure effective NTM diagnosis in the long term for efficient surveillance systems, and also to develop a point-of-care test for NTM identification in limited-resource settings.

## Data Availability

The datasets analyzed during the current study to generate the results are not publicly available, but can be provided by the corresponding author on reasonable request.

## Abbreviations

TB: Tuberculosis
NTM: Non-tuberculous Mycobacteria
MBTC: *Mycobacterium* Tuberculosis Complex
LPA: Line Probe Assay
MGIT: *Mycobacterium* Growth Indicator Tube
HIV: Human Immunodeficiency Virus
DTC: Diagnosis and Treatment Centers
CPC: Centre Pasteur du Cameroun
DST: Drug Susceptibility Testing
TB-NRL: Tuberculosis-National Reference Laboratory
